# Periodontitis-related salivary microbiota aggravates Alzheimer’s disease via gut-brain axis crosstalk

**DOI:** 10.1080/19490976.2022.2126272

**Published:** 2022-09-29

**Authors:** Jiangyue Lu, Shuang Zhang, Yuezhen Huang, Jun Qian, Baochun Tan, Xueshen Qian, Jia Zhuang, Xihong Zou, Yanfen Li, Fuhua Yan

**Affiliations:** Nanjing Stomatological Hospital, Medical School of Nanjing University, Nanjing, China

**Keywords:** Periodontitis, Alzheimer’s disease, saliva, gut microbiota, gut-brain axis, inflammation

## Abstract

The oral cavity is the initial chamber of digestive tract; the saliva swallowed daily contains an estimated 1.5 × 10^12^ oral bacteria. Increasing evidence indicates that periodontal pathogens and subsequent inflammatory responses to them contribute to the pathogenesis of Alzheimer’s disease (AD). The intestine and central nervous system jointly engage in crosstalk; microbiota-mediated immunity significantly impacts AD via the gut-brain axis. However, the exact mechanism linking periodontitis to AD remains unclear. In this study, we explored the influence of periodontitis-related salivary microbiota on AD based on the gut-brain crosstalk in APP^swe^/PS1^ΔE9^ (PAP) transgenic mice. Saliva samples were collected from patients with periodontitis and healthy individuals. The salivary microbiota was gavaged into PAP mice for two months. Continuous gavage of periodontitis-related salivary microbiota in PAP mice impaired cognitive function and increased β-amyloid accumulation and neuroinflammation. Moreover, these AD-related pathologies were consistent with gut microbial dysbiosis, intestinal pro-inflammatory responses, intestinal barrier impairment, and subsequent exacerbation of systemic inflammation, suggesting that the periodontitis-related salivary microbiota may aggravate AD pathogenesis through crosstalk of the gut-brain axis. In this study, we demonstrated that periodontitis might participate in the pathogenesis of AD by swallowing salivary microbiota, verifying the role of periodontitis in AD progression and providing a novel perspective on the etiology and intervention strategies of AD.

## Introduction

Periodontitis is one of the most common oral inflammatory disease caused by dysbiosis of dental biofilm.^1,2^ Studies have demonstrated that periodontitis is closely associated with a variety of inflammatory systemic diseases such as diabetes, cardiovascular diseases, and obesity, etc.^3–5^ In addition, a functional connection between periodontitis and Alzheimer’s disease (AD) has been established in the past decade.^6–8^ The pathogenic microorganisms of periodontitis and subsequent inflammatory responses may have significant implications for AD development.^9,10^ However, the exact mechanisms linking periodontitis and AD require further exploration.

The oro-digestive translocation of oral pathobionts resulting from periodontitis can induce inflammatory responses at extra-oral sites linked to intestinal dysbiosis and gut-associated systemic inflammation.^11,12^ The oral cavity is the initial part of the digestive tract; humans ingest an estimated 1.5 × 10^12^ oral bacteria per day from swallowed saliva.^13^ The salivary microbiota composition in patients with periodontitis significantly differs from that in healthy individuals.^14^ Besides, studies have shown a positive correlation between salivary and subgingival levels of periodontal pathogens, such as *Porphyromonas gingivalis* and *Treponema denticola*.^15,16^ The transmission of oral species to gut is a frequent and continuous process that can be increased under pathological conditions.^17^ A clinical study reported altered gut microbiota in patients with periodontitis, with an increased abundance of the phyla *Firmicutes, Proteobacteria, Euryarchaeota*, and *Verrucomicrobia* compared to healthy individuals.^18^ Moreover, in a previous study, we found that periodontitis-related salivary microbiota disordered the gut microbiota, exacerbated the systemic immune response, and worsened colitis in mice.^19^ Together, these studies indicate that the salivary microbiota may significantly participate in the mechanisms of periodontitis in systemic inflammatory diseases.

The intestine and central nervous system (CNS) engage in bidirectional communication with each other, and microbiota-induced immunity exerts significant effects on neurodegenerative diseases via the gut-brain axis.^20^ Recent studies have presented compelling evidence that the pathogenesis and progression of AD are correlated with dysbiosis of the gut microbiota, systemic inflammation, and neuroinflammation.^21,22^ Gut microbiota changes in AD include a reduction in diversity of bacterial species and an increase in the abundance of pro-inflammatory bacteria that correlate with the cerebrospinal fluid biomarkers of AD.^23^ The loss of commensal bacteria in gut can affect immune responses and disrupt colonization resistance to potential pathogens.^24^ Intestinal barrier dysfunction associated with gut microbiota dysbiosis has been observed in elderly people and AD mouse models; it triggers increased inflammation levels in blood circulation and subsequently in the CNS.^25,26^ Notably, both periodontitis and AD are associated with intestinal dysbiosis and systemic inflammation. Gut-mediated immune responses resulting from the oro-digestive translocation of periodontopathic bacteria may participate in the deterioration of AD. Animal experiments in mice have shown that oral administration of *Porphyromonas gingivalis* induces cognitive impairment and gut dysbiosis.^27^ However, the effect of periodontitis-related salivary microbiota on AD has not yet been explored, which can be expected to have more significant clinical implications on AD than a single species of periodontal pathogenic bacteria.

In this study, we aimed to investigate the impact of periodontitis-related salivary microbiota on AD by gavage of the salivary microbiota from patients with periodontitis and healthy individuals in an APP^swe^/PS1^ΔE9^ (PAP) mouse model of AD. This study reinforced the role of the periodontitis-related salivary microbiota in the crosstalk between the gut and brain during AD progression.

## Result

### Participants’ information

A total of 53 subjects were enrolled, including 27 patients with periodontitis and 26 healthy controls (HCs) without periodontitis (Table 1). The inclusion and exclusion criteria are presented in Table S1. There were no significant differences in age or sex between the two groups (Table 1). All 27 patients had periodontitis stage III or IV, representing periodontitis severity (Table S2).

**Table 1.** Characteristics of study participants.

	Periodontitis n = 27	Healthy controls n = 26	*p* value
Age in years, mean ± SD	32.6 ± 6.8	31.0 ± 6.0	NS^a^
Male, % (n)	63.0 (17)	65.4 (17)	NS^b^
Female, % (n)	37.0 (10)	34.6 (9)	NS^b^
Periodontitis stage			
III, % (n)	66.7 (18)		
IV, % (n)	33.3 (9)		

^a^T-test. ^b^ Chi-square test.

### Alterations of salivary microbiota composition in patients with periodontitis

**Figure 1.**
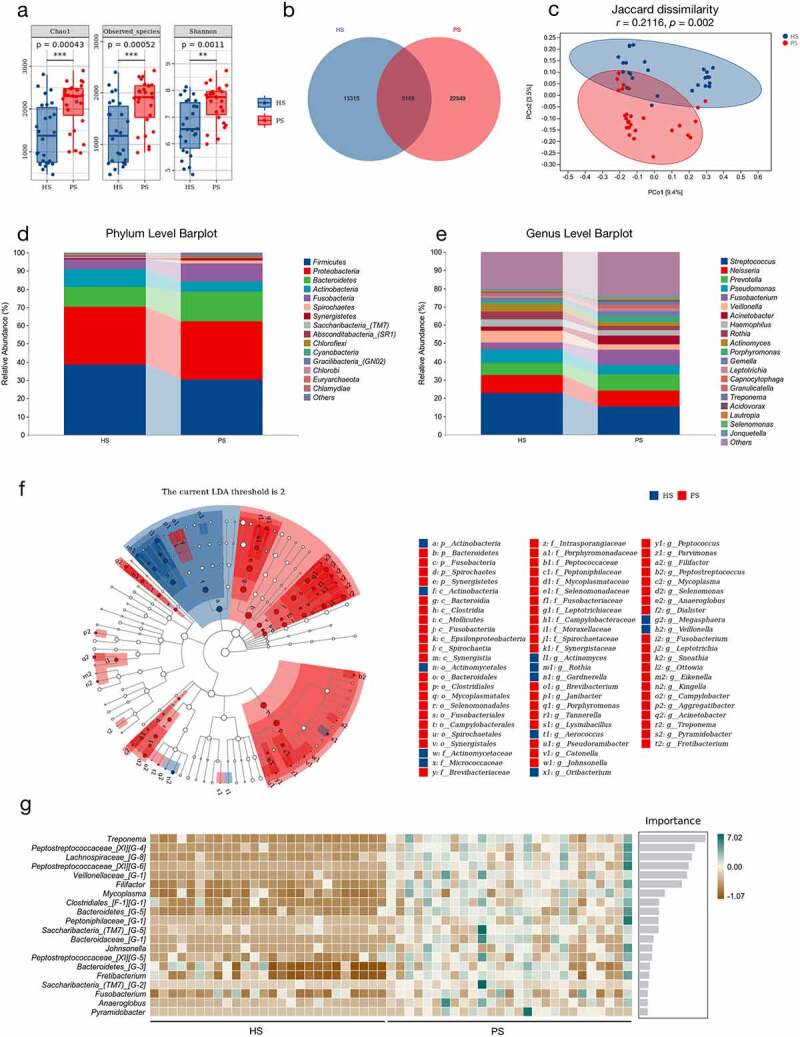
The differences of salivary microbiota in patients with periodontitis and healthy controls according to the 16S rRNA data. (a) Alpha-diversity indices of bacterial species in periodontitis saliva (PS) and healthy saliva (HS), including Chao1, Observed species, and Shannon index. Each box plot represented the median, interquartile range, minimum, and maximum values (n = 26–27). (b) Venn diagram indicating amplicon sequence variants (ASVs) between HS and PS. (c) Principal coordinate analysis (PCoA) of salivary microbiota on the basis of Jaccard dissimilarity. ANOSIM, *r* = 0.2116, *p* = .002. (d-e) Relative abundance of microbiota at the phylum (d) and genus (e) levels between two groups. (f) LDA Effect Size (LEfSe) Cladograms showing differences in the bacterial taxa between HS and PS (LDA > 2). (g) Random forest analysis indicating the importance score for genus between HS and PS. *p < .05, **p < .01, ***p < .001.

The microbial composition of saliva samples was assessed using 16S ribosomal RNA (rRNA) sequencing. Various alpha-diversity indices (Chao1, Observed species, and Shannon) indicated that the species richness of microbiota in periodontitis saliva (PS) was significantly increased compared with that of healthy saliva (HS) (Figure 1a). Similarly, the Venn diagram of bacteria showed that 17,380 out of 22,549 amplicon sequence variants (ASVs) were unique to the PS group (Figure 1b). Principal coordinate analysis (PCoA) based on Jaccard dissimilarity revealed that the beta-diversity value could be used to clearly discriminate between PS and HS (ANOSIM, *r*= 0.2116, *p* = .002) (Figure 1c). Additionally, the overall composition of the salivary microbiota was further analyzed at the phylum and genus levels (Figure 1(d,e)). The phylum *Bacteroidetes* showed a tendency to increase in abundance, and *Firmicutes* decreased in the PS group compared to the HS group. Periodontal pathogens such as *Treponema, Porphyromonas*, and *Fusobacterium*, were enriched in periodontitis saliva according to the random forest and linear discriminant analysis effect size (LEfSe) results (Figure 1(f,g)). These results suggested that periodontitis can lead to significant changes in the oral salivary microbiota.

### Periodontitis-related salivary microbiota exacerbates the anxiety and cognitive impairment in PAP mice

**Figure 2.**
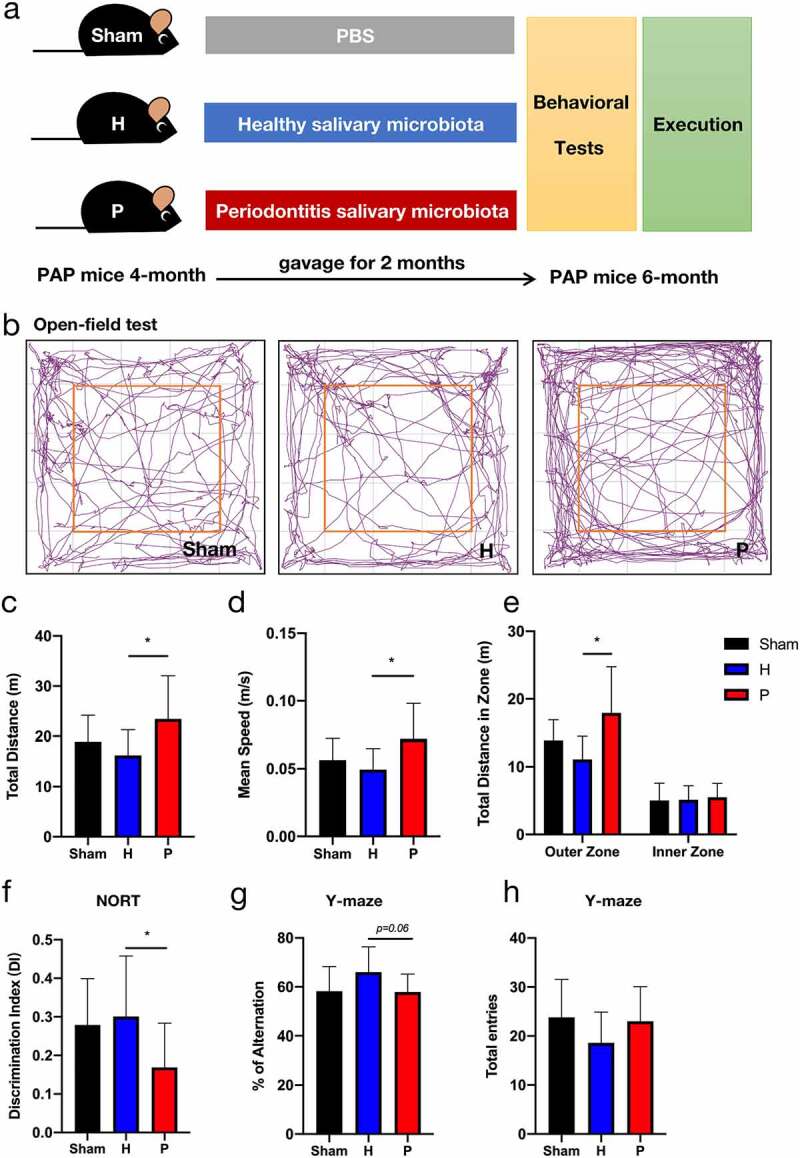
Anxiety degree and cognitive impairment in groups of PAP mice after gavaging for two months. (a) Design of the animal experiments. (b-e) The open-field test included representative tracking images of movement in 5 min (b), total distance traveled (c), mean speed (d), and total distance traveled in outer and inner zones (e). (f) Discrimination index (DI) in the new object recognition test (NORT). (g-h) Percentage of spontaneous alternation (g) and the number of total entries (h) in Y-maze task. Data were presented as the mean ± SD (n = 10), *p < .05, **p < .01. H, healthy; P, periodontitis.

To explore the influence of periodontitis on AD, salivary microbiota from the patients with periodontitis and HCs was used to gavage PAP mice (Figure 2a). We conducted three behavioral tests to evaluate the degree of anxiety and cognitive impairment in PAP mice. In the open field test, the PAP mice in the periodontitis (P) group showed a faster mean speed, longer total distance traveled, and greater distance traveled in the outer zone than the healthy (H) group (Figure 2b-e), indicating an increased level of anxiety. We then found that the mice in the P group spent the least time exploring the novel object among the experimental groups (Figure 2f), displaying the impairment of long-term cognitive ability. In the Y-maze test, short-term learning and memory impairments were exacerbated in the P group versus the H group (Figure 2g), with no obvious difference in the number of total entries (Figure 2h). These data showed that continuous gavage of the periodontitis-related salivary microbiota aggravated anxiety and cognitive impairment in PAP mice.

### Periodontitis-related salivary microbiota increases β-amyloid (Aβ) accumulation and neuroinflammation in PAP mice

**Figure 3.**
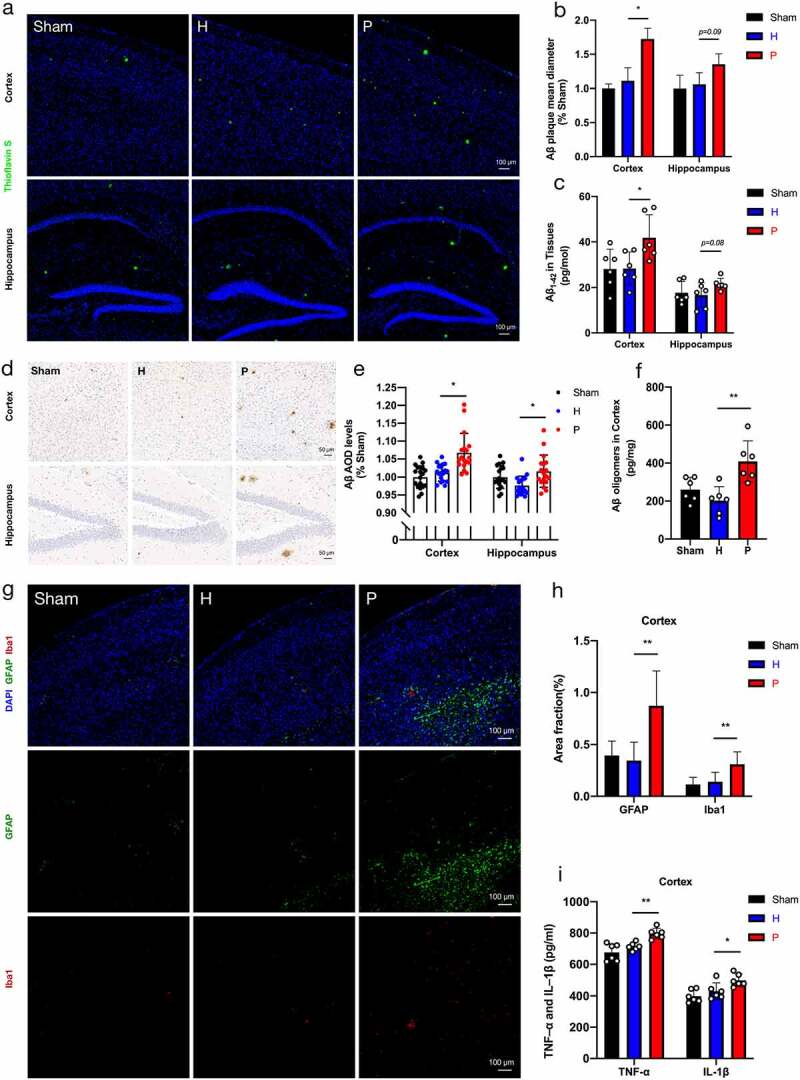
β-amyloid (Aβ) accumulation and neuroinflammation in groups of PAP mice. (a) Representative images displayed Aβ plaque stained by Thioflavin S. Scale bar = 100 µm. (b) Quantification of Aβ plaque diameter in the cortex and hippocampus (n = 3). (c) Aβ_1-42_ in cortex and hippocampus by ELISA detection (n = 6). (d-e) Representative images of Aβ immunohistochemistry (d) and quantification of Aβ immunopositivity in the cortex and hippocampus (e) (n = 3 areas in the region of interest from 6 mice per group). Scale bar = 50 µm. (f) Levels of Aβ oligomers in cortex detected by ELISA (n = 6) (g) Representative images of astrogliosis (GFAP) and microgliosis (Iba1). Scale bar = 100 µm. (h) Area fractions of GFAP and Iba1 in the cortex of mice (n = 3). (i) The expressions of TNF-α and IL-1β in cortex using ELISA detection (n = 6). These data were presented as the mean ± SD, *p < .05, **p < .01. H, healthy; P, periodontitis; AOD, average optical density.

To identify the effect of periodontitis on AD-related pathologies in brain tissue, Aβ accumulation and neuroinflammation were detected in PAP mice. We found that Aβ deposition, one of the most common AD pathologies, was higher in the P group than in the H group, according to the analyses of Thioflavin S staining (Figure 3(a,b)) and Aβ immunohistochemistry (Figure 3(d,e)). Similarly, the level of Aβ_1-42_ monomers measured by ELISA analysis exhibited the same tendency in the experimental groups (Figure 3c). In addition, Aβ oligomers, which are toxic and are believed to trigger neuropathological changes observed in AD,^28–30^ were increased in the cerebral cortex of PAP mice in the P group compared to mice in the H group (Figure 3f). Furthermore, we probed hypertrophic reactive astrocytes stained with glial fibrillary acidic protein (GFAP) and activated microglia stained with ionized calcium-binding adaptor molecule (Iba1) (Figure 3g), as the exacerbation of neuroinflammation is a well-established pathological sign of AD.^22^ Compared to the H group, both positive area fractions of GFAP and Iba1 in the cerebral cortex of PAP mice were obviously larger in the P group (Figure 3h). Moreover, ELISA analysis showed that the levels of pro-inflammatory cytokines, such as tumor necrosis factor α (TNF-α) and interleukin (IL)-1β, in the cerebral cortex of PAP mice increased significantly in the P group compared with the H group (Figure 3i). Altogether, these results revealed that periodontitis-related salivary microbiota might exacerbate AD lesions, particularly immunopathological responses in the brains of PAP mice.

### Periodontitis-related salivary microbiota intensifies intestinal inflammation and impairs intestinal barrier in PAP mice

**Figure 4.**
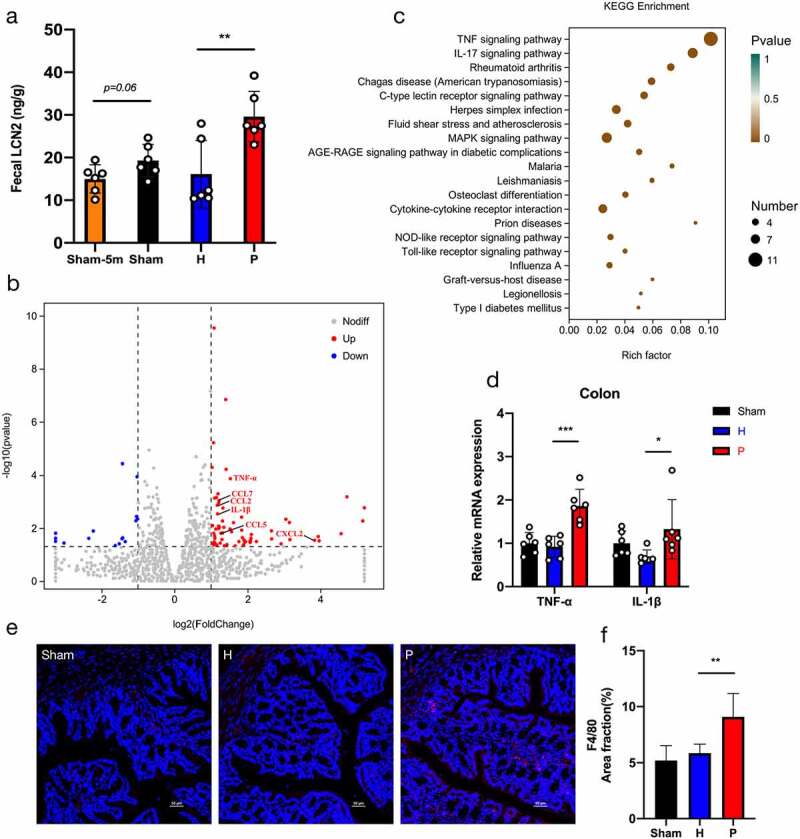
Periodontitis-related salivary microbiota intensifies intestinal inflammation in PAP mice. (a) Concentrations of fecal lipocalin-2 (LCN2) detected by ELISA in groups of Sham-5 m (at 5 months of age), Sham, H and P (n = 6). (b) Visualization of differentially expressed genes (DEGs) in the colon tissue of mice in P group compared with H group (n = 3). (c) Bubble diagram of the KEGG enrichment analysis based on DEGs between P and H groups. The horizontal axis represented the gene ratio and the size of dots represented the number of genes in the KEGG term. (d) Relative mRNA expressions of TNF-α and IL-1β in the colon tissue (n = 6). (e-f) Representative images of macrophages (F4/80) (e) and quantification of area fractions in the colon tissue (f) (n = 3). Scale bar = 50 µm. Data were presented as the mean ± SD, *p < .05, **p < .01, ***p < .001. H, healthy; P, periodontitis.

Adverse intestinal inflammation is associated with AD progression.^21,26^ Our experiment also verified that intestinal inflammation was exacerbated in AD development. The level of fecal lipocalin-2 (LCN2), which promotes intestinal inflammation, was higher in PAP mice at 6 months of age compared to 5 months (Figure 4a). Interestingly, fecal LCN2 levels were upregulated in the P group (Figure 4a). RNA sequencing was performed to probe the exact effect of the periodontitis-related salivary microbiota on intestinal tissue in PAP mice. The results showed that periodontitis-related salivary microbiota treatment significantly changed the overall landscape of immune response-related gene expression in the intestine, with conspicuous upregulation of differentially expressed genes (DEGs), such as TNF-α, IL-1β, CCL2, CCL5, CXCL2, and CCL7 in the colon tissue (Figure 4b). KEGG enrichment analysis based on DEGs showed that immune-related signaling pathways such as the TNF signaling pathway, IL-17 signaling pathway, and Cytokine-cytokine receptor interaction were upregulated in the P group compared with the H group, especially the TNF signaling pathway (Figure 4c). To further validate the inflammatory responses related to the TNF signaling pathway, the levels of TNF-α and IL-1β in the colon tissue of PAP mice were measured. We found that the mRNA expressions of TNF-α and IL-1β were markedly increased in the P group, particularly the level of TNF-α (Figure 4d). The secretion of TNF-α is known to be positively correlated with intestinal macrophage activity.^31^ The immunofluorescence assessment of intestinal macrophages (F4/80+) indicated that periodontitis-related salivary microbiota treatment remarkably promoted the infiltration of macrophages into colon tissue (Figure 4(e,f)). Together, these results provided evidence that intestinal pro-inflammatory responses in PAP mice were intensified by continuous exposure to the periodontitis-related salivary microbiota.

**Figure 5.**
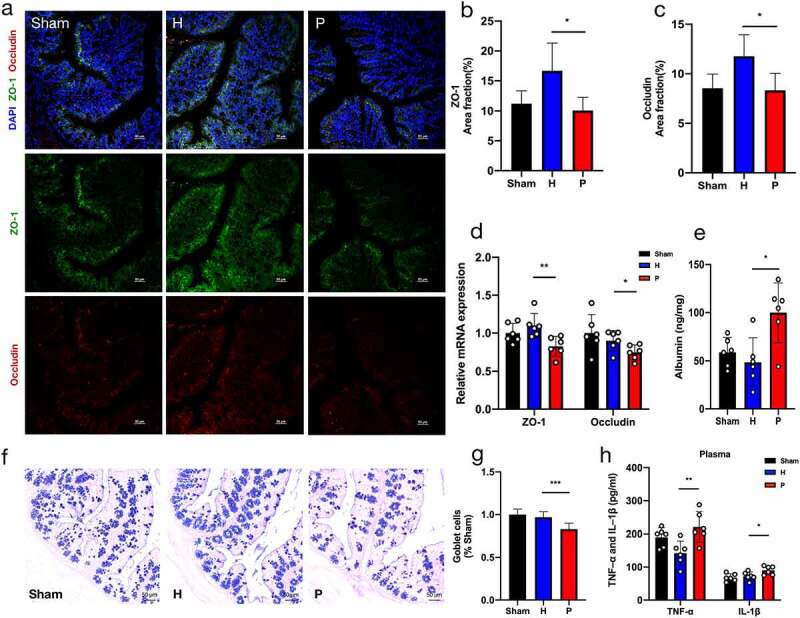
Periodontitis-related salivary microbiota impairs intestinal barrier in PAP mice. (a-c) Representative images of ZO-1 and occludin immunostaining (a) and quantification of area fractions in the colon of PAP mice (b-c) (n = 3). Scale bar = 50 µm. (d) Relative mRNA expressions of ZO-1 and occludin (n = 6). (e) Concentrations of fecal albumin detected by ELISA (n = 6). (f-g) Alcian blue and periodic acid-Schiff (AB-PAS) staining showing the distribution of goblet cells in colon (f) and the goblet cells per crypts that normalized to the Sham group (g) (n = 3). Scale bar = 50 µm. (h) The ELISA test indicating levels of TNF-α and IL-1β in plasma of mice (n = 6). Data were presented as the mean ± SD in the different experimental groups, *p < .05, **p < .01, ***p < .001. H, healthy; P, periodontitis.

Serious intestinal pro-inflammatory responses are generally associated with the impairment of the intestinal barrier.^32^ Compared with mice gavaged with healthy salivary microbiota, periodontitis-related salivary microbiota significantly reduced the expressions of tight junction-related proteins ZO-1 and occludin (Figure 5a–c), which play an essential part in maintaining the intestinal barrier. The mRNA expression levels of ZO-1 and occludin again validated these results (Figure 5d). Periodontitis-related salivary microbiota treatment also considerably increased the content of fecal albumin in PAP mice, which is recognized as a biomarker for the assessment of intestinal permeability (Figure 5e).^33,34^ Additionally, Alcian blue and periodic acid-Schiff (AB-PAS) staining showed the damage to intestinal mucus layer in the P group, with a decreased number of goblet cells in each crypt (Figure 5(f,g)). Furthermore, severe intestinal inflammation and barrier impairment may lead to increased systemic inflammation.^35,36^ ELISA analysis showed that the plasma concentrations of TNF-α and IL-1β, which reflected the level of systemic inflammation, were significantly increased in mice treated with periodontitis-related salivary microbiota compared to those in healthy mice (Figure 5h). Particularly, the intestinal pro-inflammatory responses triggered by periodontitis-related salivary microbiota exposure might induce intestinal barrier impairment and elevated systemic inflammation.

### Periodontitis-related salivary microbiota induces gut microbial dysbiosis in PAP mice

**Figure 6.**
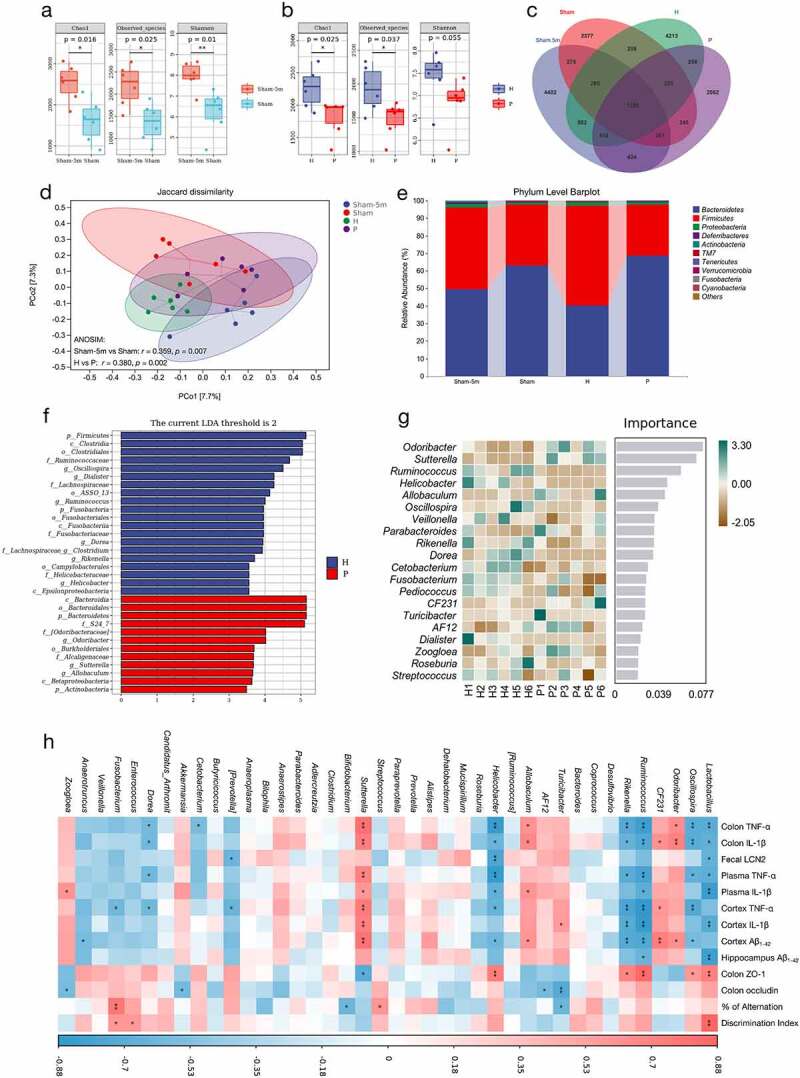
Periodontitis-related salivary microbiota alters the composition of gut microbiota in PAP mice. (a-b) Fecal microbial diversity estimated by Chao1, Observed species and Shannon index of PAP mice between the ages of 5 and 6 months in Sham group (a) and between H and P groups (b) (n = 6). (c) A Venn diagram showing the overlaps among groups. (d) PCoA of fecal microbiota on the basis of Jaccard dissimilarity and ANOSIM. Sham-5 m vs Sham, *r* = 0.359, *p* = .007; H vs P, *r* = 0.380, *p* = .002. (e) Relative abundance of microbiota at the phylum level among groups. (f-g) LEfSe analysis (g) and random forest analysis (g) indicating the fecal taxonomic differences between H and P groups. (h) Correlation analysis of top 40 microbes with AD-related parameters based on the Spearman correlation coefficient test. A color gradient from blue (negative correlation) to red (positive correlation) indicating the correlation effect. *p < .05, **p < .01. H, healthy; P, periodontitis.

Dysbiosis of the intestinal microflora is associated with peripheral inflammation and brain amyloidosis in AD.^26^ To observe the alteration of gut microbiota during AD progression, we studied the fecal flora in five-month-old PAP mice at the beginning of the Aβ pathology, compared to that at the age of 6 months when AD-related lesions were in progress. 16S rRNA analysis revealed that fecal microbial alpha-diversity was significantly decreased at the age of 6 months (Figure 6a), representing a reduction in diversity and richness of the gut microbiota. In addition, the Venn diagram of bacteria and beta-diversity conducted using PCoA based on Jaccard dissimilarities revealed that the gut microbiota composition of PAP mice varied at different stages of AD (Figure 6(c,d)).

Next, to determine the effect of periodontitis-related salivary microbiota on the gut microbiota in PAP mice, we tested the fecal flora in the H and P groups after gavage of salivary microbiota for two months. A significant decline in alpha-diversity of the gut microbiota was also observed in the P group compared with the H group (Figure 6b). The Venn diagram and PCoA of bacteria showed that salivary microbiota from the patients with periodontitis and HCs caused different alternations in the gut microbiota composition (Figure 6(c,d)). At the phylum level, the amount of *Bacteroidetes* increased by 28.33%, while that of *Firmicutes* decreased by 27.66% in the P group versus the H group (Figure 6e). A similar tendency was also observed between the PAP mice in the Sham group at the ages of 5 and 6 months (Figure 6e). At the genus level, *Odoribacter, Sutterella, Ruminococcus, Helicobacter, Allobaculum, Oscillospira, Rikenella*, and *Dorea* were the most representative differential microbiota between the P and H groups, according to the LEfSe results and random forest analysis (Figure 6(f,g)).

To identify the specific bacterial taxa that might be associated with AD development, Spearman’s correlation analysis of the top 40 microbes with AD-related parameters in PAP mice was performed (Figure 6h). Among these bacterial genera, *Lactobacillus, Oscillospira, Ruminococcus, Rikenella*, and *Helicobacter* were negatively correlated with most AD phenotypes. Moreover, *Sutterella, Allobaculum*, and *Odoribacter* displayed strong positive correlations with the AD-related parameters (Figure 6h). Notably, the relative abundance of *Sutterella* was significantly increased in the P group versus the H group (*p* < .01) (Figure S1A), whereas that of *Lactobacillus* was reduced in the P group, although no significant difference between groups was found (Figure S1B). The above results indicated that the intestinal microbiota disturbance caused by periodontitis-related salivary microbiota might be related to the aggravation of AD lesions.

## Discussion

**Figure 7.**
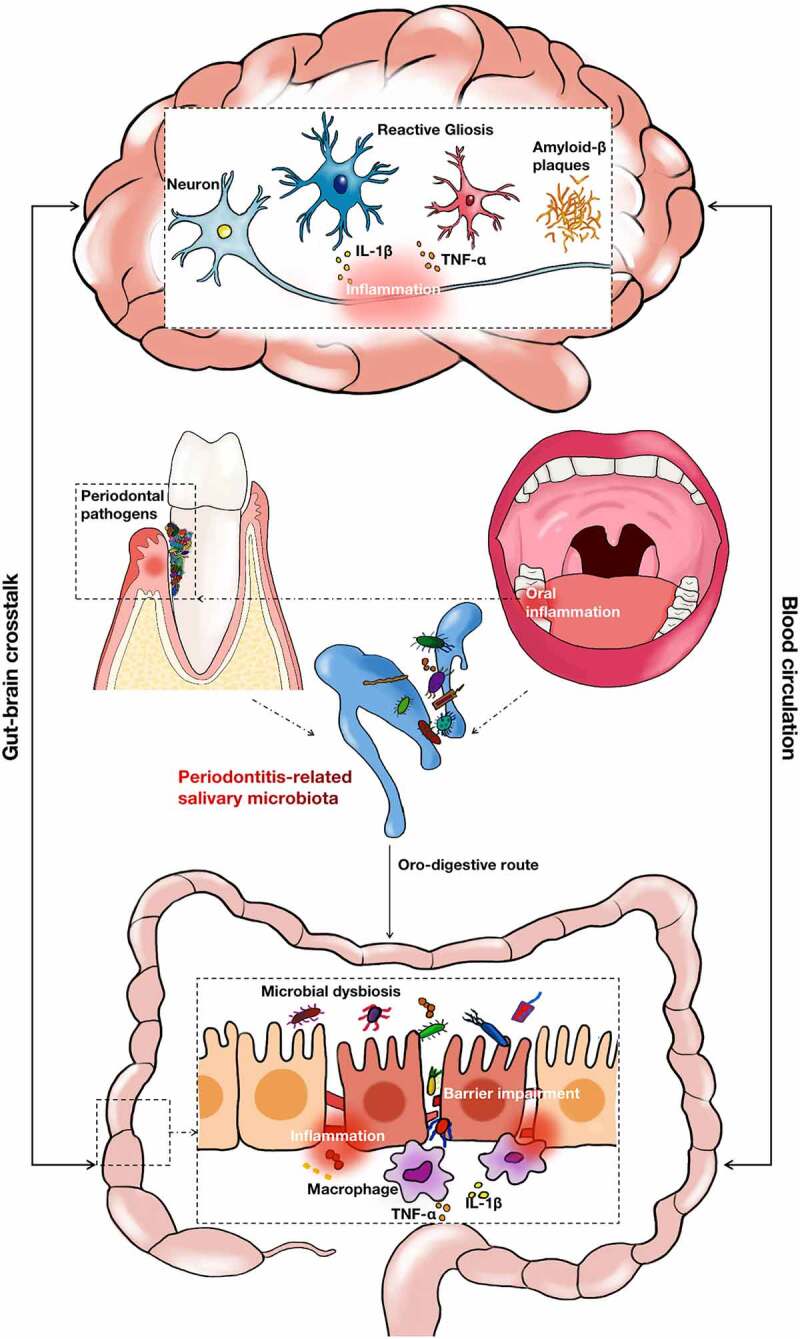
The schematic diagram for this study. Periodontitis led to significant changes in the composition of salivary microbiota. The oro-digestive translocation of periodontitis-related salivary microbiota induced gut microbial dysbiosis, intestinal proinflammatory responses and intestinal barrier impairment. These gut-associated pathogenesis may interact mutually with the brain lesions of AD through the blood circulation system.

The purpose of this study was to explore the potential role of periodontitis-related salivary microbiota in AD pathogenesis. This study revealed that the salivary microbiota composition in patients with periodontitis was significantly changed compared with healthy individuals, with the enrichment of periodontal pathogens such as *Treponema, Porphyromonas*, and *Fusobacterium*. Continuous gavage of periodontitis-related salivary microbiota in PAP mice resulted in gut microbial dysbiosis, intestinal pro-inflammatory responses, and intestinal barrier impairment, subsequently leading to the exacerbation of systemic inflammation. The above gut-associated lesions were consistent with impaired cognitive function, increased Aβ accumulation and neuroinflammation in PAP mice, suggesting that the periodontitis-related salivary microbiota may aggravate AD pathogenesis through bidirectional gut-brain communication (Figure 7).

The oro-digestive translocation of oral pathobionts resulting from periodontitis can induce gut-mediated systemic inflammation.^11,27^ In PAP mice, periodontitis-related salivary microbiota caused alterations in colonic gene expression patterns associated with pro-inflammatory responses, and most of these genes were involved in the TNF signaling pathway. TNF-α can be secreted by activated intestinal macrophages and is critical for the initiation of the nuclear factor kappa-B (NF-κB) transduction pathway, affecting gene transcription, regulating downstream inflammatory response, and apoptosis.^37,38^ Moreover, by remodeling tight junctions in epithelial tissues and downregulating the expression of occludin, TNF-α can lead to epithelial barrier loss and increased intestinal permeability.^32,39^ Accordingly, in our experiment, the PAP mice in the P group showed reduced expression of intestinal tight junction-related proteins and damage to the intestinal mucus layer, indicating intestinal barrier impairment caused by the periodontitis-related salivary microbiota. Furthermore, the increased plasma concentrations of TNF-α and IL-1β reflected systemic inflammation, which might be attributed to gut-associated lesions.

Under pathological conditions, the oral microbiota can colonize in the gut and disrupt the intestinal gut microbiota.^40,41^ Since intestinal inflammation and gut microbiota dysbiosis have been considered prominent features in the progression of AD,^26,42^ periodontitis-related salivary microbiota might produce cascade effects on the intestine of PAP mice. In our study, the periodontitis-related salivary microbiota treatment reduced gut microbial richness and diversity, a ubiquitous indicator of disease-associated dysbiosis.^43–45^ In addition, an increased abundance of *Bacteroidetes* was observed in mice gavaged with periodontitis-related salivary microbiota compared to that in healthy mice, in accordance with gut microbiota alterations in patients with AD.^23^ The phylum *Bacteroidetes* consists of a diverse group of gram-negative commensal bacteria in the intestine and is more predominant than *Firmicutes* in the elderly.^46,47^ According to Spearman’s correlation analysis with AD-related parameters, *Lactobacillus, Sutterella, Oscillospira, Ruminococcus, Rikenella*, and *Helicobacter* at the genus level might be crucial for gut-brain bidirectional communication in AD pathogenesis. *Lactobacillus* is a probiotic that ameliorates brain inflammation by participating in gastric vagus nerve activity.^48^ Compared with the healthy control, the mice in the P group exhibited decreased *Lactobacillus* abundance, although not significantly; this may be due to the limitation of gavage time. In addition, *Sutterella* was significantly enriched after gavage with the periodontitis-related salivary microbiota in our study. Although the role of *Sutterella* in AD remains unclear, clinical studies have found that this microbe can be identified in fecal samples and intestinal biopsies from individuals with colitis and autism; it has been suspected of having a pro-inflammatory capacity in the intestine.^49,50^

Gut-brain crosstalk plays a principal part in the occurrence and development of AD, mainly via the pathways of the immune system, microbial metabolites, vagus nerve, and enteric nervous system, all of which are highly complex and interconnected.^51^ In our study, cognitive impairment, increased Aβ accumulation, and brain inflammation were observed in PAP mice treated with periodontitis-related salivary microbiota. Notably, brain inflammation was strongly associated with intestinal inflammation in PAP mice in our experiment, indicating that immune communication between the gut and brain may play an essential part in the progression of periodontitis-aggravating AD lesions. In particular, changes in the gut microbiota may interact with the intestinal immune response. The results of our study show that periodontitis-related salivary microbiota exposure disturbed intestinal homeostasis by altering the gut microbiota and affecting the intestinal immune status in the AD mouse model. Previous studies have demonstrated that the microbiota reacts with local intestinal immune cells and releases pro-inflammatory cytokines into the blood circulation system, producing systemic effects beyond the gastrointestinal tract.^52,53^ Thus, the systemic inflammation observed in PAP mice gavaged with periodontitis-related salivary microbiota may be caused by altered gut microbiota, intestinal inflammation, and loss of epithelial barrier integrity. Furthermore, reactive glial activation, which modifies neuroinflammation, can be triggered by aberrant levels of pro-inflammatory cytokines in the plasma through the blood-brain barrier (BBB).^54,55^ In this regard, the activation of astrocytes and microglia was observed in mice in the P group, which simultaneously increased the levels of inflammatory cytokines in the cerebral tissue. In addition, intestinal inflammatory mediators can also reach the CNS through the lymphatic system or vagus nerve in the gastrointestinal tract.^56,57^ However, the mechanisms that translate the peripheral immune response induced by the periodontitis-related salivary microbiota into AD-related neuroinflammation need to be investigated.

Recently, the relevance and importance of the oral microbiota in systemic diseases have received much focus.^12,41,58–60^ In this study, we demonstrated that periodontitis might participate in the pathogenesis of AD via the swallowing of salivary microbiota, suggesting the significance of oral examination and treatment in the prevention of AD. Potential mechanisms include disturbance of intestinal homeostasis, aggravation of systemic inflammation, and crosstalk between the gut microbiota and the CNS. Based on these findings, this study proves the role of periodontitis in AD progression and provides a novel perspective on an etiology and intervention strategies for AD. However, further investigations are needed to explore how periodontitis-related salivary microbiota disrupts gut homeostasis and exacerbates brain lesions. For example, specific components or metabolites of microbiota may play a crucial role in this oral-gut-axis.

## Materials and methods

### Collection and treatment of human saliva samples

All participants in this study were recruited from the Nanjing Stomatological Hospital, Medical School of Nanjing University. Ethical approval was obtained from the committee of Nanjing Stomatological Hospital, Medical School of Nanjing University (Ethics NO, 2019NL-008KS), and a written informed consent was signed by all participants upon enrollment. Saliva sample collection involved each volunteer slowly spitting the unstimulated saliva into a sterile centrifuge tube every 2 min until the total saliva volume exceeded 4 mL. The collected saliva samples were centrifuged at 1000 rpm for 10 min; the sediments were removed and mixed with the same volume (w/v) of phosphate buffer solution (PBS) containing 20% glycerol (G5516, Sigma), and then stored at −80°C. When used, different samples from the same group were pooled first, centrifuged at 4000 × g for 10 min, suspended in PBS, and gavaged to specific pathogen-free (SPF) mice (200 µL per mouse).

### Animal model

Four-month-old male PAP transgenic mice (purchased from HFK Bioscience Co., Ltd., Beijing, China) were maintained under SPF conditions at the animal laboratory of Nanjing Agricultural University. After acclimatization for one week, all PAP mice were divided into three groups (n = 10 per group), and each group was gavaged every other day for two months as follows: PBS (sham group), salivary microbiota from the HCs (H group), and salivary microbiota from the patients with periodontitis (P group). Behavioral assays were conducted during the light cycle after gavage for two months. At the ages of 5 and 6 months, fresh feces (0.1 g) of each mouse were separately collected in sterile centrifuge tubes and stored at −80°C. In addition, the cortex, hippocampus, plasma, and colon samples of the mice were collected after the behavioral experiments and stored at −80°C until further use. All animal experiments were performed in accordance with the laboratory animals ethical committee of the Nanjing Agricultural University (Ethics NO, PZW2021020) and followed the Animal Research: Reporting of In Vivo Experiments (ARRIVE) guidelines.

### Behavioral assays

#### Open field test

The open-field test assessed anxiety-like behaviors in mice.^61^ The open-field arena (40 × 40 cm) was averagely divided into 25 blocks. The outer zone was composed of 16 blocks, and the inner zone was composed of 9 blocks. Mice were gently placed in the same position and allowed to explore the open-field arena for 5 min. The total distance traveled, mean speed, and distance traveled in the inner and outer zones were recorded and analyzed using the ANY-maze (Stoelting Co., USA).

#### Novel object recognition test (NORT)

NORT was used to evaluate the long-term cognitive abilities of the animals.^62^ For two consecutive days, the PAP mice were allowed to get acquainted with two identical objects placed in the apparatus (40 × 40 cm) for 5 min. On the third day, one of the two familiar objects was replaced with a novel object, and the PAP mice were gently placed in the apparatus and allowed to explore the objects for 5 min. A camera was used to record the exploration time of mice for each object. Within 5 min, the exploration times of the familiar object (F) and novel object (N) were recorded and analyzed. The exploration was defined as sniffing (within 1 cm), biting, or pawing. Distinction index (DI) was calculated as the formula: DI = (N − F/N + F) ×100%.

#### Y-maze

Y-maze was used to evaluate the short-term spatial learning and memory of mice.^63^ The PAP mice were allowed to explore the Y-maze device, which consisted of three arms at equal angles (40 × 8 × 15 cm) for 5 min. Alternation behavior was defined as the consecutive entries into three different arms. The % of Alternation was calculated according to the formula: [the number of alternations/(the total number of arm entries − 2)] × 100%.

### Thioflavin S staining

The brain hemispheres of PAP mice were fixed in 4% paraformaldehyde for 2 days, dehydrated in graded ethanol, embedded in paraffin wax and cut into 5 µm sagittal sections. For fibrillar Aβ staining, brain sections of mice were incubated in thioflavin S (S19293, Yuanye, Shanghai, China) solution for 8 min at room temperature, then washed with ethanol and distilled water. The slides were mounted in a fluorescence mounting medium (G1401, Servicebio, Wuhan, China), dried in the dark for 2 h, and protected with coverslips. The size of Aβ plaque was determined by manually tracing its diameter. A total of six microscopic fields of each section were examined, including three fields in the cerebral cortex and three fields in the hippocampus.

### Aβ immunohistochemical analysis

For Aβ immunohistochemical staining, 5 µm-thick brain sections of PAP mice were cut and deparaffinized with graded alcohols. After repairing antigens with the citric acid antigen repair buffer (G1202, Servicebio, China), sections were washed in PBS, blocked in serum solution, and then incubated with the anti-β-Amyloid (D3D2N) mouse antibody (1:800) (15,126, Cell Signaling Technology, USA) according to the manufacturer’s instructions. Then the corresponding secondary antibody was used, followed by 3,3’-diaminobenzidine (G1211, Servicebio, China) staining. In each brain section, three representative fields of cerebral cortex or hippocampus were photographed and the average optical density (AOD) was analyzed using ImageJ software (National Institutes of Health, USA).

### ELISA

The cerebral cortex and hippocampus tissues of PAP mice were homogenized in radio immunoprecipitation assay lysis buffer (P0013B, Beyotime, China) containing protease inhibitors (ST506, Beyotime, China), sonicated briefly, lysed on ice for 30 min with gentle agitation, and then centrifuged at 14,800 × g for 20 min at 4°C to remove debris. Aliquots of the lysates were tested for Aβ_1-42_ monomers and Aβ oligomers, using the Human/Rat Beta Amyloid (42) ELISA kit (290–62601, Wako, Japan) and Mouse Aβ Oligomer ELISA kit (AB6094, Abmart, China), respectively, according to the manufacturer’s protocols. Plasma and brain tissue IL–1β and TNF–α were determined using the Mouse IL-1β (EMC001b, NeoBioscience, China) and TNF-α (EMC102a, NeoBioscience, China) ELISA kits in accordance with the manufacturer’s protocols. In addition, feces of PAP mice were homogenized at 50 mg/mL in sterilized PBS, and large debris were removed by brief pulsed centrifugation. The fecal suspension was centrifuged at 14,800 × g for 15 min at 4°C to remove sediments, and the levels of fecal LCN2 and albumin were assayed using the Mouse LCN-2/neutrophil gelatinase-associated lipocalin (NGAL) Quantikine ELISA kit (MLCN20, R&D Systems, USA) and Mouse Albumin ELISA kit (E13878m, Cusabio, China).

### Immunofluorescence

Immunofluorescent staining was conducted as described previously.^19^ Antibodies are presented in Table S3. After immunofluorescent staining, Nikon Ti confocal laser microscopy (Nikon, Japan) was used to visualize the colon and brain sections. Three microscopic fields of view from three PAP mice per group were used to quantify the fractions of positively stained areas with analytic software ImageJ (National Institutes of Health, USA).

### AB-PAS staining

The colon tissues of mice were fixed in 4% paraformaldehyde for 2 days, dehydrated, embedded in paraffin wax, and sectioned at 5 µm. AB-PAS (G1049, Servicebio, Wuhan, China) was used to stain the sections in accordance with the manufacturer’s protocols, and the goblet cells per crypt were statistically analyzed and normalized.

### Quantitative real-time polymerase chain reaction

RNA from mouse colon tissue was extracted using an RNA Fast Tissue Kit (DP451, Tiangen Company, Beijing, China) in accordance with the manufacturer’s protocols, and cDNA was synthesized using Prime Script RT Master Mix (RR036A, TaKaRa, Japan). The primer pairs used are presented in Table S4.

### 16S rRNA sequencing and data analysis

Illumina NovaSeq sequencing of salivary and fecal microbiota was performed by Personal Biotechnology Company (Shanghai, China). After DNA extraction, the V3–V4 region of bacterial 16S rRNA was amplified using microbiome primers 338 F and 806 R. The compositions of salivary and fecal microbiota were determined using the Illumina NovaSeq-PE250 platform and QIIME2 bioinformatics analysis. Taxonomy was assigned to ASVs against the Greengenes database. Alpha-diversity indices, including Shannon, Chao1, and observed species were analyzed using the ASVs table in QIIME2 and visualized as box plots. Beta-diversity analysis was conducted using Jaccard metrics and visualized using PCoA. LEfSe was used to identify differentially abundant taxa at the genus level across the groups.

### Colonic transcriptome analysis

Transcriptomic analysis of the mouse colon tissue was performed (Personal Biotechnology, Shanghai, China). The sequencing library was sequenced using an Illumina NovaSeq 6000 platform. Read count values for each gene were generated using high-throughput sequence (HTSeq) statistics and fragments per kilobase per million (FPKM). The DEGs were then analyzed by DESeq with screened conditions as follows: P < .05 and > 2-fold change. The ClusterProfiler software was used to conduct KEGG enrichment analysis of the DEGs.

### Statistical analysis

All data were shown as the mean ± standard deviation (SD). The correlation between bacterial taxa and AD-related parameters was evaluated using Spearman’s correlation coefficient test. Differences between the mean values of the two groups were assessed by independent sample t-tests. One-way analysis of variance was performed to calculate the mean values between more than two groups. Statistical analyses were conducted with SPSS Statistics version 26.0 (IBM Corp., USA).

## Supplementary Material

Supplemental Material

## Data Availability

This is an Open Access article distributed under the terms of the Sequence Read Archive (PRJNA818859), which permits unrestricted use, distribution, and reproduction in any medium, provided the original work is properly cited. https://www.ncbi.nlm.nih.gov/bioproject/PRJNA818859
