# Supplementary material for: Periodontitis-related salivary microbiota aggravates Alzheimer’s disease via gut-brain axis crosstalk
Source: Gut Microbes. 2022 Sep 29;14(1):2126272. doi: 10.1080/19490976.2022.2126272 (PMC9542625; doi:10.1080/19490976.2022.2126272)
Supplement: Supplemental Material [file KGMI_A_2126272_SM8847.docx]

**Supplemental data：**

**
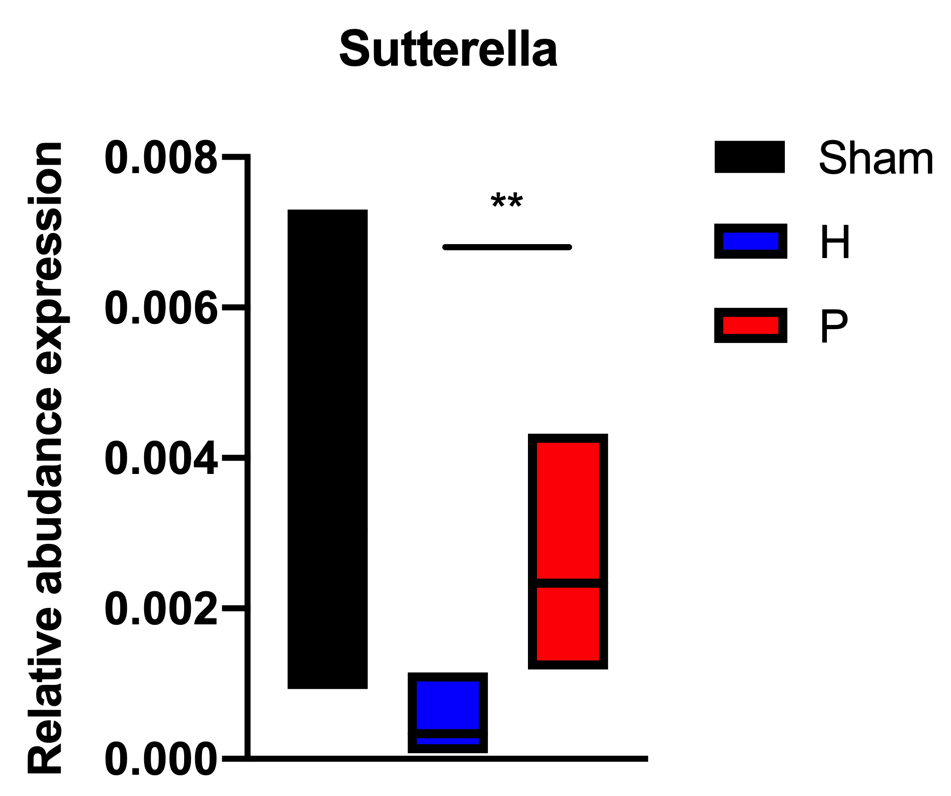

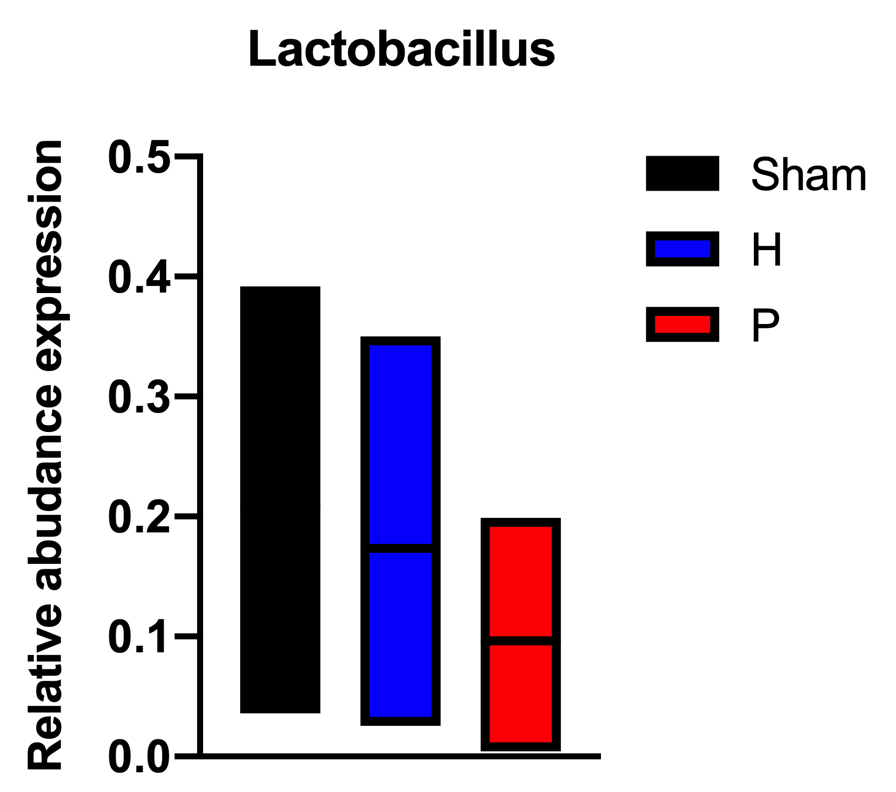
 A B**

Figure S1. (A and B) Relative abundance of *Sutterella* and *Lactobacillus* in Sham, H and P groups. Each box represented the median, interquartile range, minimum, and maximum values. **p* < 0.05. ** *p* < 0.01. H, healthy; P, periodontitis.

**Supplementary Table 1.** The inclusion and exclusion criteria of participants.

| **Inclusion criteria:** |
| --- |
| 1) Aged 18-60 years;  2) At least 18 teeth in the mouth;  3) Without systemic diseases;  4) Patients with clinical diagnosis of periodontitis stage III or IV, and there were > 30% sites have attachment loss and bone resorption in the whole teeth. |
| **Exclusion criteria:** |
| 1) Received periodontal treatment within 6 months;  2) Long-term use of antibiotics within 6 months;  3) Received nonsteroidal anti-inflammatory drugs therapy within 3 months;  4) Comorbidity that may influence with the patient's safety: diabetes, immune deficiency, gastrointestinal diseases, cardiovascular and cerebrovascular diseases, blood diseases and so on;  5) Other serious oral diseases;  6) Pregnancy or lactation;  7) Smokers (>5 PCS/day);  8) BMI≥30kg/m^2^. |

**Supplementary Table 2.** The diagnosis of patients with periodontitis.

| **Number** | **Diagnosis** |
| --- | --- |
| P1 | Periodontitis (stage III) |
| P2 | Periodontitis (stage III) |
| P3 | Periodontitis (stage III) |
| P4 | Periodontitis (stage III) |
| P5 | Periodontitis (stage III) |
| P6 | Periodontitis (stage IV) |
| P7 | Periodontitis (stage IV) |
| P8 | Periodontitis (stage III) |
| P9 | Periodontitis (stage III) |
| P10 | Periodontitis (stage III) |
| P11 | Periodontitis (stage IV) |
| P12 | Periodontitis (stage III) |
| P13 | Periodontitis (stage III) |
| P14 | Periodontitis (stage IV) |
| P15 | Periodontitis (stage IV) |
| P16 | Periodontitis (stage III) |
| P17 | Periodontitis (stage III) |
| P18 | Periodontitis (stage IV) |
| P19 | Periodontitis (stage III) |
| P20 | Periodontitis (stage IV) |
| P21 | Periodontitis (stage III) |
| P22 | Periodontitis (stage III) |
| P23 | Periodontitis (stage IV) |
| P24 | Periodontitis (stage III) |
| P25 | Periodontitis (stage III) |
| P26 | Periodontitis (stage IV) |
| P27 | Periodontitis (stage III) |

**Supplementary Table 3.** Antibodies for immunofluorescence.

| Protein | Supplier | Catalog number | Dilution |
| --- | --- | --- | --- |
| Iba1 | Servicebio | GB131105 | 1:200 |
| GFAP | Servicebio | GB12096 | 1:200 |
| ZO-1 | Servicebio | GB111981 | 1:4000 |
| Occludin | Servicebio | GB111401 | 1:5000 |
| F4/80 | Servicebio | GB11027 | 1:200 |

**Supplementary Table 4.** Primers sequences.

| **Name** | **Forward Primer** | **Reverse Primer** |
| --- | --- | --- |
| *M. Actin* | 5’-AGAGGGAAATCGTGCGTGAC-3’ | 5’-CAATAGTGATGACCTGGCCGT-3’ |
| *M. TNF-α* | 5’-TCTTCTCATTCCTGCTTGTGG-3’ | 5’-GAGGCCATTTGGGAACTTCT-3’ |
| *M. IL-1β* | 5’-CAACCAACAAGTGATATTCTCCATG-3’ | 5’-GATCCACACTCTCCAGCTGCA-3’ |
| *M. ZO-1* | 5’-CCACCTCTGTCCAGCTCTTC-3’ | 5’-CACCGGAGTGATGGTTTTCT-3’ |
| *M. Occludin* | 5’-GGGCCATCTCAACTCCTGTA-3’ | 5’-AGAAGGGCTGACGGGTAAAT-3’ |
